# Coronary CT Angiography in Managing Atherosclerosis

**DOI:** 10.3390/ijms16023740

**Published:** 2015-02-09

**Authors:** Joachim Eckert, Marco Schmidt, Annett Magedanz, Thomas Voigtländer, Axel Schmermund

**Affiliations:** Cardioangiologisches Centrum Bethanien, Im Prüfling 23, D-60389 Frankfurt, Germany; E-Mails: m.schmidt@ccb.de (M.S.); a.magedanz@ccb.de (A.M.); t.voigtlaender@ccb.de (T.V.); a.schmermund@ccb.de (A.S.)

**Keywords:** atherosclerosis, coronary plaques, coronary computed-tomography angiography (CCTA), coronary calcium, cardiac events

## Abstract

Invasive coronary angiography (ICA) was the only method to image coronary arteries for a long time and is still the gold-standard. Technology of noninvasive imaging by coronary computed-tomography angiography (CCTA) has experienced remarkable progress during the last two decades. It is possible to visualize atherosclerotic lesions in the vessel wall in contrast to “lumenography” performed by ICA. Coronary artery disease can be ruled out by CCTA with excellent accuracy. The degree of stenoses is, however, often overestimated which impairs specificity. Atherosclerotic lesions can be characterized as calcified, non-calcified and partially calcified. Calcified plaques are usually quantified using the Agatston-Score. Higher scores are correlated with worse cardiovascular outcome and increased risk of cardiac events. For non-calcified or partially calcified plaques different angiographic findings like positive remodelling, a large necrotic core or spotty calcification more frequently lead to myocardial infarctions. CCTA is an important tool with increasing clinical value for ruling out coronary artery disease or relevant stenoses as well as for advanced risk stratification.

## 1. Background

Recent developments of CT scanners have improved accuracy especially regarding the visualization of the coronary arteries. A better spatial and temporal resolution makes it possible to scan the heart and the coronary arteries free of motion and to detect vascular plaques and stenoses. Still, heart rates below 60–65/min are preferable to achieve high quality images with a low radiation exposure using prospective ECG (electrocardiographic)-gating. Common nomenclature distinguishes between different types of plaque: calcified, noncalcified and predominant calcified or predominant noncalcified [1]. Calcified plaques are visualized and quantified by CT scans without injection of contrast agent (calcium scanning). For detecting different types of plaque as well as determining possible coronary stenoses, intravenous contrast agent must be injected prior to the scan (CT-angiography, CTA).

## 2. Coronary Plaque Morphology and Pathophysiology

On the basis of the CT images, coronary plaques are classified as calcified and noncalcified or as “mixed” plaques containing both aspects. Pathophysiologically, subendothelial lipoprotein retention triggers inflammatory responses via macrophages and T-cells with chronic maladaptive progression of atherosclerotic lesions [2]. Looking at plaques on a cellular basis, early atherosclerotic changes can be classified into 3 types [3] which reflect microscopic changes like accumulation of macrophages (type I) and which are already seen in infant arteries. Later, fatty streaks, foam cells and deposits of lipid inside smooth-muscle cells can be found (type II). These lesions tend to start to develop in puberty. Type III lesions mark the border where these microscopic changes become visible to the eye. Macroscopic changes begin, and the so-called “atheroma” is formed. Advanced lesions can again be classified into 3 types (types IV–VI) [4]. Type IV lesions encompass the lipid core which is called atheroma. As soon as fibrous tissue grows the lesion is classified type V (“fibroatheroma”). If a thrombus or hemorrhage develops on the atheroma or fibroatheroma the lesion is regarded “complicated” (type VI) and, hence, patients can become symptomatic. Lesions IV and V can be asymptomatic due to maintenance of the vessel diameter. Glagov *et al.* first described adaptive changes of arterial size in the course of plaque formation [5]. The entire vessel grows with increasing plaque volume so that the lumen diameter is maintained. Furthermore, a frequent pathology seen in myocardial infarctions due to plaque rupture is the thin cap fibroatheroma (TCFA), which is characterized by a necrotic core covered by a fibrous cap measuring <65 µm [6]. Even though the classifications cannot be directly compared, the TCFA corresponds to a subgroup of the Stary type V lesion. Speckled calcification can be visualized in the majority of ruptured plaques. TCFA seems to be the precursor lesion of plaque rupture. It is frequently associated with expansive remodeling. These changes cannot be detected in invasive angiography because the vessel wall is invisible and only the lumen, which may appear normal, is displayed. Coronary CT-angiography (CCTA) may fill this diagnostic gap, since changes of the vessel wall can directly be visualized.

## 3. Coronary Calcification

**Figure 1:**
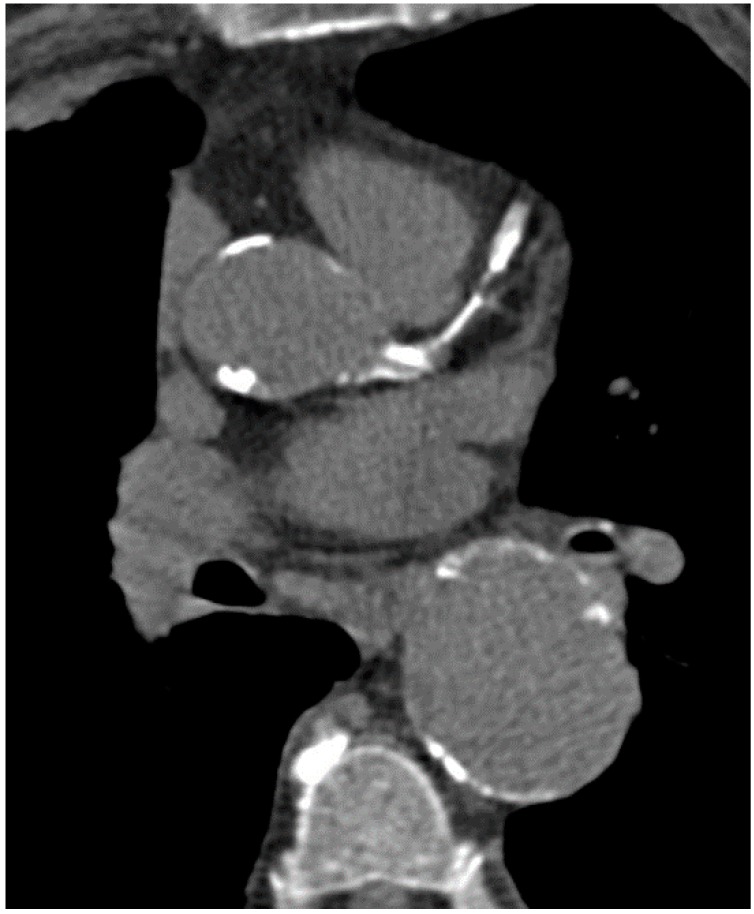
Native calcium scan with severe calcification of left main, left anterior descending (LAD) and the aorta.

Coronary artery calcification (CAC) is a frequent pathology seen in CT scans (Figure 1). The amount of calcium is quantified using the Agatston-Score [7]. It is correlated with the extent of atherosclerotic plaque burden [8]. In most patients presenting with acute coronary syndromes or sustaining sudden cardiac death, calcifications in the coronary artery wall can be detected [9,10]. A high amount of calcium, however, does not necessarily correlate with angiographic luminal stenoses, nor is there a fixed relationship with vulnerability of plaques [11]. *Vice versa*, a lack of coronary calcium makes stenotic lesions unlikely, but it is not possible to definitely rule out coronary stenoses [12,13].

There are still debates on the mechanisms of coronary artery calcification. Studies could show that calcification is not a mere passive response to injury but an active process similar to bone formation [14,15]. This process already starts in the second decade of life [16]. Mostly, calcifications are part of atherosclerotic changes, share the same risk factors, and can predominantly be found in advanced lesions [17].

The amount of calcium is influenced by gender, ethnicity and age [18]. Different data exist concerning the possible individual modification of coronary calcium. Lifestyle changes and aggressive medical therapy (especially with “statins”) might slow the progress of calcification [19,20]. Interestingly, recent data show that the progression of calcification is mainly driven by genetic conditions and to a minor extent by classical risk factors such as hypertension or LDL cholesterol [21,22]. It is, however, important that although progression of calcification seems to be inevitable this does not hold true for the clinical outcome and adverse cardiac events of patients on lifestyle changes or medication for risk-factor modification.

## 4. Clinical Implication and Prognosis of Coronary Artery Calcium

Studies have demonstrated that cardiovascular events are low and the overall prognosis is good in the absence of coronary calcifications [23]. Coronary calcium scoring in combination with assessment of the Framingham Score in asymptomatic people can improve risk stratification especially in individuals with risks between 10% and 19% in 10 years according to the Framingham Score [24]. High calcium scores are associated with future cardiovascular events and worse survival outcome. Cardiovascular risk increases proportionally to the amount of calcium and is highest with Agatston-Scores above 400. An annual progression of more than 15% enhances the risk of myocardial infarctions [17,19,25]. Patients after myocardial infarctions have higher CAC progressions than subjects who remained event-free [26]. Positive predictive values of CAC progression as a marker of risk are, however, low [17]. Repeated CAC scans can therefore not be recommended as a control of adequate medical therapies or lifestyle changes. Single calcium scores are recommended in asymptomatic persons with intermediate risk (Framingham risk score 10%–20%) as support for clinical decisions whether to start aggressive medical therapy. In high or low risk populations, CAC scoring does not necessarily add relevant information.

## 5. Coronary CT Angiography

**Figure 2:**
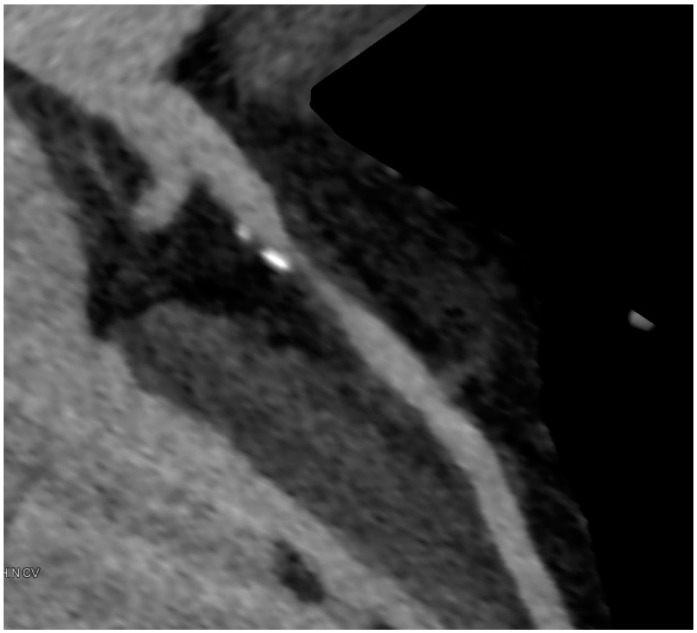
Predominantly noncalcified plaque with high-grade stenosis of LAD.

**Figure 3:**
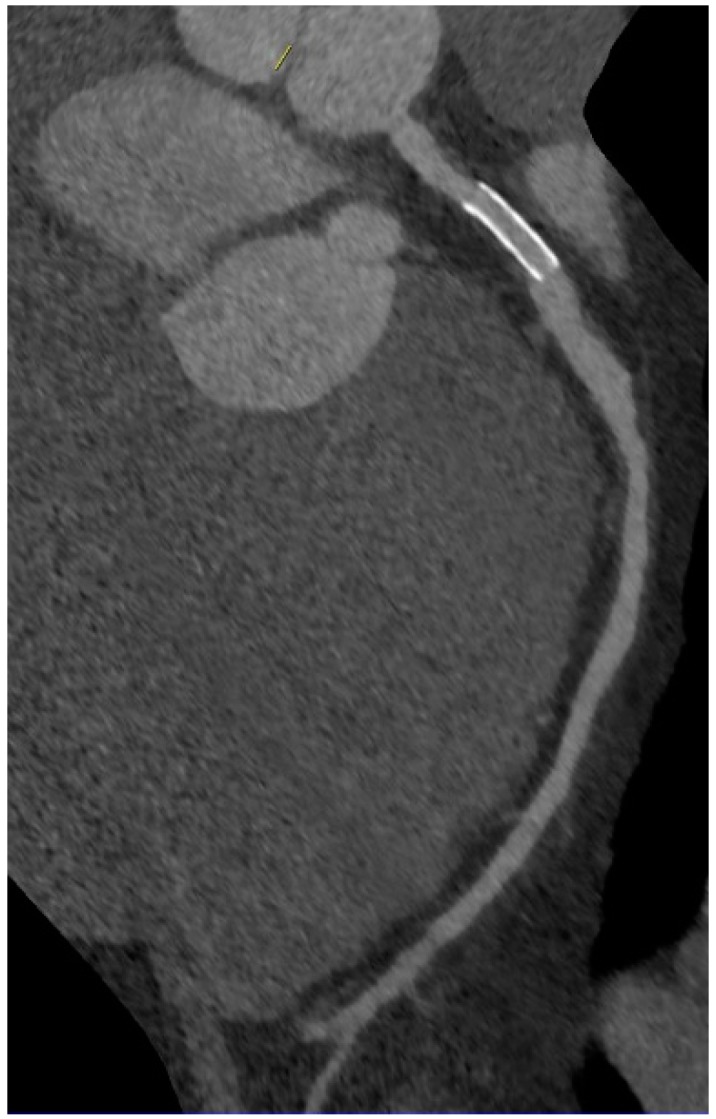
LAD after revascularization with a patent drug eluting stent showing a very good result 18 months after implantation.

**Figure 4:**
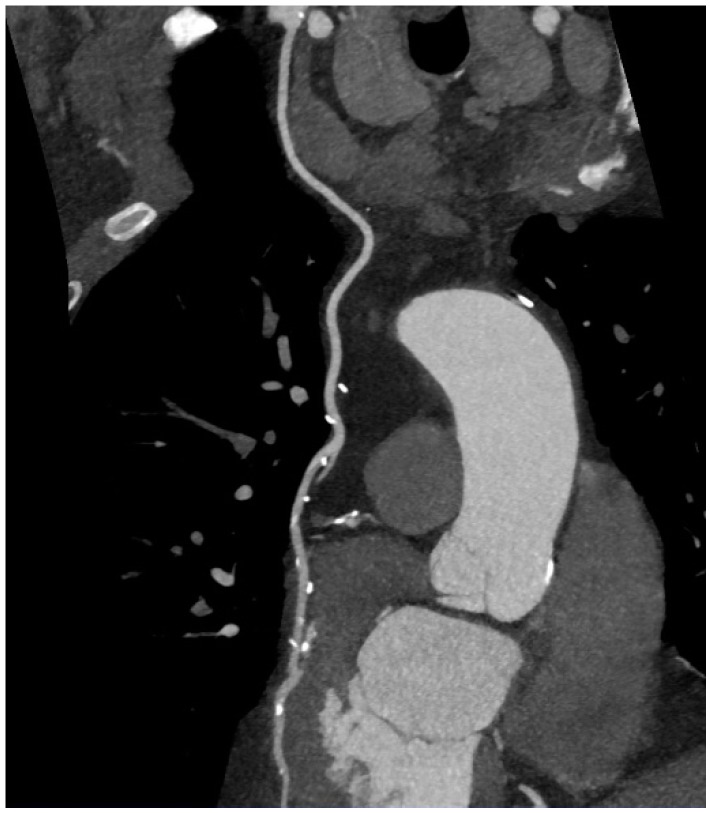
Patent right mammary artery bypass graft (free transplant with end-to-side anastomosis on left mammary artery) with anastomosis on obtuse marginal branch.

For calcium scoring, a native CT scan is sufficient. To gain information on coronary stenoses and plaque morphology, contrast media (50–100 mL) must be injected and the scan timed in the phase of maximal contrast enhancement. In contrast to invasive coronary angiography (ICA), CCTA offers the advantage of visualizing the vessel wall. Thus, it is possible to detect atherosclerotic lesions despite a preserved vessel lumen as well as lesions causing a coronary stenosis (Figure 2), even in revascularized patients (Figure 3 and Figure 4).

For clinical purposes, CCTA performs best in individuals who are at low to intermediate risk of coronary artery disease (CAD) [27]. For high-risk individuals, the diagnostic performance of CCTA is lower; patients frequently need ICA afterwards due to suspected high-grade stenoses in CCTA or severe calcifications.

Using the latest CT scanners (at least 2 × 128 slices), CCTA can be performed with a radiation exposure of <1 mSv. High pitch spiral mode with iterative reconstruction is able to visualize the whole heart in a single diastole with excellent image quality [28,29,30]. To obtain images with low radiation exposure and little motion artifacts, patients’ heart rate should be <60–65/min. Beta blockers are often administered prior to the scan.

## 6. Imaging of Coronary Plaques and Stenoses

**Figure 5:**
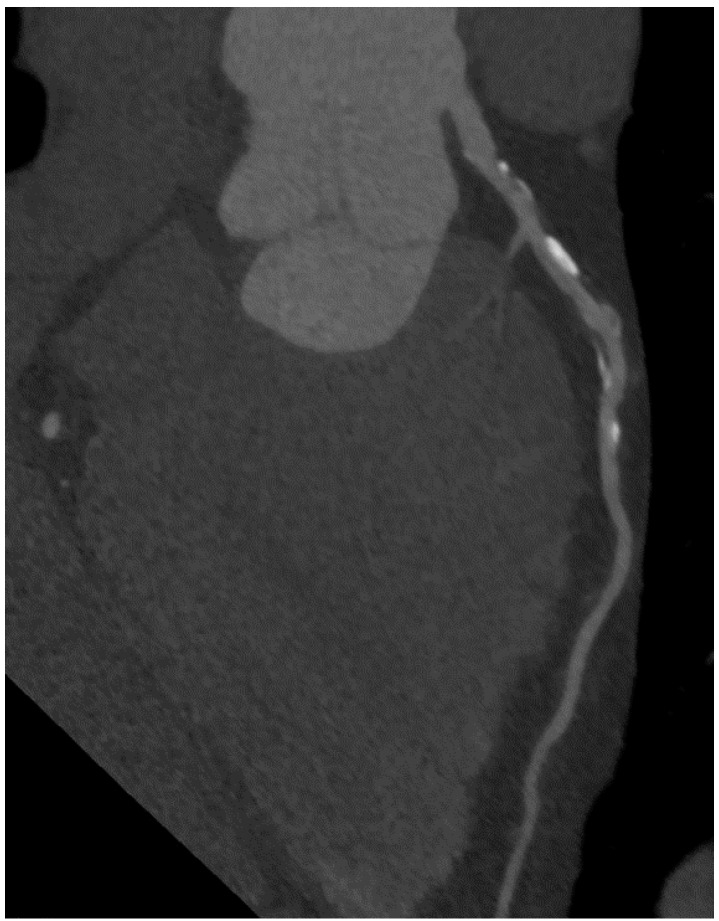
Calcified and noncalcified plaques in LAD.

**Figure 6:**
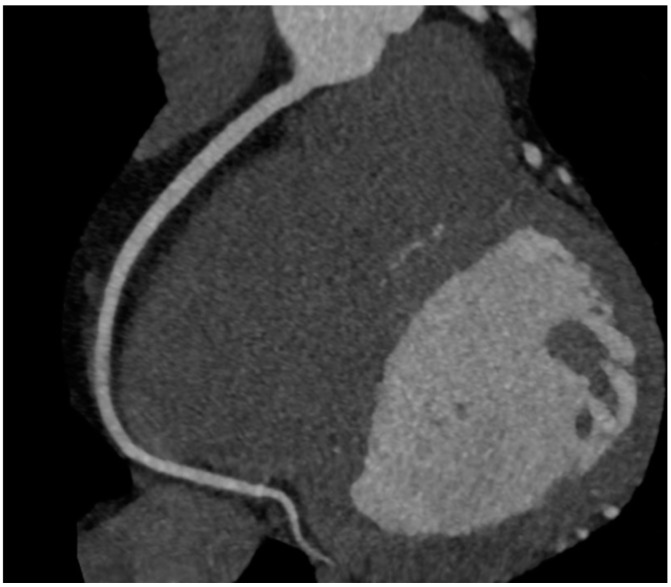
Normal right coronary artery.

When performing CCTA in patients with intermediate risk for CAD, a substantial portion of the patients show coronary plaques (Figure 5). Hausleiter *et al.* assessed 161 patients of whom almost 30% had noncalcified plaques; most had both noncalcified and calcified plaques. In this group, 6% had plaques without any calcification [31]. Several studies compared the diagnostic accuracy of detecting coronary artery stenoses compared to invasive angiography [32], some additionally with intravascular ultrasonography (IVUS) [33,34,35]. Sensitivity for detection of plaques range above 90%, negative predictive values approach 100% in patients with low to intermediate probabilities of CAD. CCTA is a reliable method, especially for ruling out relevant plaques and stenoses in coronary arteries (Figure 6). One major limitation is a reduced ability to reliably quantify the degree of stenoses [36] which is the reason for lower positive predictive values and specificity due to the fact that stenoses tend to be overestimated in CCTA especially in calcified lesions. Specificity ranges between 64% and 87%, depending on patient characteristics such as obesity or calcification [32,35,37]. A recent meta-analysis comprised 42 studies in which CCTA was compared to IVUS for detection of any plaques. Sensitivity and specificity were 93% and 92%, respectively [34]. Furthermore, imaging artifacts can lead to misinterpretation. Most of the existing studies were, however, performed using 64-slice CCTA. Technology has remarkably improved in the last decade so that dual-source scanners with 2 × 128 slices and more are the technical standard at present. In a meta-analysis by Voros *et al*., it could be demonstrated that sensitivity improves from 84% to 94% when images are obtained with 64-slice scanners compared to 16-slice scanners [38]. Still, different attenuation values inside the same plaques (fibrous, lipid-rich, necrotic and calcified) make the classification and reproducibility of lesions challenging.

Cheng *et al.* demonstrated that visual detection of plaque presence is reproducible [39]. Intraobserver, interobserver and interscan variability were excellent, but large differences in agreement existed regarding total plaque volume. The reason is probably the problem of quantifying small coronary plaques by CCTA due to technical limitations in spatial resolution. Moderate reproducibility of plaque burden and degree of coronary stenoses was also reported by Leber *et al.* using 64-slice CT scanners [36,40]. Interobserver variability depends on image quality. Pflederer *et al*. showed that in the left anterior descending coronary artery (LAD), where image quality was best, interobserver variability was significantly lower than in the left circumflex (LCX) or right coronary artery (RCA) (17% in LAD *versus* 32% RCA) [41].

A commonly used standardized score for quantification of coronary calcification is the Agatston-Score [7]. A standardized, reproducible tool for quantification of noncalcified plaques does not exist. One important reason for that is the limited spatial resolution of CCTA that makes small plaques difficult to detect. Furthermore, noncalcified plaques can show a wide range of attenuation values due to differences in morphology. An automated quantification using a special software to assess minimal lumen area, plaque burden, percentage luminal stenosis and degree of remodeling was used by Boogers *et al*. in 51 patients [42]. Plaque quantification was feasible and reproducible, and significant correlations could be demonstrated for all parameters. Minimal lumen area was, however, underestimated, and lumen area stenosis overestimated compared to IVUS, especially in calcified lesions.

Furthermore, CCTA is a helpful planning tool for revascularization of chronic total coronary occlusions (CTO). Rolf *et al.* could show that CCTA prior to percutaneous coronary intervention (PCI) could significantly improve success rates of the intervention in 30 patients [43]. Three-dimensional images derived from CCTA guided the advance of the guide wire during the intervention and could thus lead to a success rate of 90% *versus* 63% in the control group without CT. Another study with 100 patients could demonstrate significantly fewer complications, such as coronary perforations, but no improvement in success [44]. Severe coronary calcification seems to be an independent predictor of failure of revascularization of a CTO lesion [45].

## 7. Qualitative Plaque Characterization

IVUS is considered the gold standard for *in vivo* plaque quantification and characterization [46]. Normal intimal thickness of healthy young subjects measures 0.15 mm [47], below the spatial resolution of CCTA, which is 0.24–0.3 mm for the latest generation of CT scanners. The vessel wall becomes thickened in atherosclerotic lesions which makes it a diagnostic target for non invasive CT evaluation.

Attenuation values inside noncalcified plaques vary according to histological findings. Fibrotic tissue is associated with higher CT density whereas a necrotic core is negatively correlated to density, although with a wide range of overlap [33,48]. Contrast flow rates and concentration inside the vessel as well as microcalcifications often seen around the necrotic core affect density measurements [49,50]. Furthermore, slice thickness and convolution kernels hamper reproducibility of density measurements. Dual-energy CT might enhance the differentiation between the necrotic core and fibrous tissue, however, with a loss of temporal resolution [51].

Atherosclerotic lesions can lead to acute coronary events and death. The morphological characterization of plaques being prone to complications is of eminent interest, and some studies have been reported on aspects of plaque morphology in the context of acute coronary syndromes. Characteristics of ruptured plaques include expansive remodeling, a large necrotic core, thin cap fibroatheroma (TCFA) and macrophage infiltration [52]. Motoyama *et al*. demonstrated that in patients with acute coronary syndromes ACS, CT can identify plaques showing expansive remodeling, low atheroma attenuation values, and spotty calcifications [53]. In another study, 1059 patients underwent CCTA; 45 of these patients showed expansive remodeling and low attenuation plaques [54]. Twenty-two percent of the patients harboring both pathologies developed myocardial infarction in the follow-up period. On the other hand, only four of 820 patients with neither sign had a cardiac event. Hoffmann *et al.* suggested that culprit lesions tend to have greater noncalcified areas, whereas largely calcified plaques indicate more stability [55].

A special pathology in atherosclerotic lesions is the so-called “napkin-ring-sign” which has a high specificity and positive predictive value for advanced lesions [56]; it can be visualized by using CCTA. The napkin-ring-sign is characterized by a plaque core with low attenuation surrounded by a rim-like area of higher attenuation, potentially representing TCFA. Recently, Otsuka *et al.* could demonstrate that the presence of the napkin-ring-sign is strongly associated with acute coronary syndromes [57].

Another interesting approach of detecting vulnerable plaques might be ^18^F-sodium fluoride and ^18^F-FDG uptake diagnosed by PET-CT. Dweck *et al.* demonstrated that ^18^F-NaF uptake was significantly higher in individuals with coronary atherosclerosis (defined by calcium score > 0) in contrast to subjects without (calcium score 0) [58]. Uptake of ^18^F-NaF seems to be related to inflammation and active calcification with similarities to bone metabolism. Recently, evaluation of patients with myocardial infarction could show that >90% of the patients had increased uptakes in the culprit lesions [59]. In other plaques with increased uptake, high-risk factors such as expansive remodelling, microcalcifications, and a larger necrotic core could be seen on IVUS. It has yet to be demonstrated that increased uptake will translate into future cardiac events.

## 8. Hemodynamic Relevance of Angiographic Stenoses

Frequently, intermediate stenoses (30%–70%) of coronary arteries are detected on CCTA and it is not evident if these lesions cause ischemia. For intermediate stenoses diagnosed on invasive angiography it is recommended to perform Fractional Flow Reserve (FFR) measurements to assess the functional relevance. De Bruyne *et al.* demonstrated that patients having lesions with FFR of less than 0.8 benefit from revascularization whereas stenoses with FFR of more than 0.8 should be treated conservatively with medical therapy alone [60]. An FFR < 0.8 is thus considered to cause ischemia. Sensitivity for diagnosing high grade stenoses on CCTA is excellent; specificity is, however, poor due to false positive results because of overestimation of stenoses [61]. There was a weak correlation between significant coronary lesions on CCTA and ICA combined with FFR < 0.75; diagnostic accuracy was only 49%. Relying solely on the visual aspect might lead to unnecessary revascularizations and be potentially harmful. Hence, the concept of measuring FFR(CT) noninvasively by CCTA was perceived during the last years. Min *et al.* could show in a multicenter trial that diagnostic accuracy for FFR(CT) was superior to CCTA alone although specificity was still poor [62]. FFR(CT) measurements seem to be reproducible [63], and can be calculated from the normal CCTA dataset without additional image acquisition by using special equations of fluid dynamics [64].

## 9. CCTA in the Emergency Department

CCTA can be used to rule out relevant CAD in the emergency department for patients presenting with symptoms such as angina without signs of myocardial infarction (ST(segment)-elevation on ECG, positive cardiac enzymes). Frequently, patients presenting with chest pain are admitted to hospital, or stay in the emergency department for many hours. One trial showed a significant reduction of time to diagnosis from 15 h in the control group to 3.4 h in the CCTA group [65]. Both approaches were safe, but CCTA appeared cost effective, and patients who had a CT scan required less subsequent diagnostic workup for recurrent chest pain symptoms.

The ROMICAT-Trial described an excellent sensitivity (100%) of diagnosing CAD in patients presenting with chest pain at low to intermediate pretest probability [66]. Fifty percent of all patients had no CAD at all. Patients were followed-up for two years regarding major adverse cardiac events (MACE) [67]. Patients with no CAD in CCTA had no risk for MACE in the following two years whereas risk was 4.6% in nonobstructive CAD and 30.3% in obstructive CAD. A limitation was that almost 10% of patients were lost to follow-up.

CCTA seems to be a useful diagnostic tool with good safety in the early triage of patients in the emergency department.

## 10. Prognostic Data of CCTA

**Figure 7:**
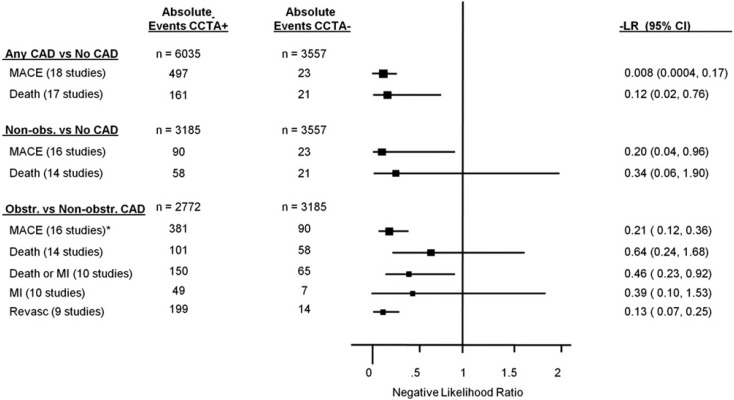
Pooled lifetime risk (LR) for major adverse cardiac events (MACE), death, death or myocardial infarctions (MI), MI and revascularization stratified by Coronary CT Angiography (CCTA) findings. Reproduced from [77] with permission from Hulten, *J. Am. Coll. Cardiol.*; published by Elsevier, 2011.

As for CAC many studies evaluated the prognostic implication of CCTA in symptomatic patients (Figure 7). Al-Mallah *et al.* followed up 8627 patients with suspected CAD concerning outcomes of death and myocardial infarction [68]. CCTA results added discriminatory power to the Agatston-Score regarding outcomes. This additional value was highest in patients with moderate calcium scores (Agatston 1–100). There is strong evidence supported by many trials that individuals without any signs of CAD in CCTA have an excellent prognosis [69,70,71,72,73]. Rates of MACE approached 0% in the years of follow-up in the studies. CCTA has not only incremental value over Calcium-Scoring but also over routine risk factors of cardiovascular disease [71,74,75,76].

On the other hand, individuals with signs of CAD on the images can be stratified regarding the risk of cardiovascular events according to different findings. Ahmadi *et al*. examined 3499 symptomatic patients of which 1102 had nonobstructive CAD; these patients were followed-up for 10 years [74]. Among the patients with plaques, event-free survival was best for patients with calcified plaques (98.6%) and decreased in mixed plaques (96.7%) and further decreased in non-calcified plaques (90.4%). Mortality rose proportionally to the amount of diseased vessels (1-, 2- or 3-vessel disease). Hou *et al.* did a follow-up on 5007 patients for myocardial infarction, death or coronary revascularization (MACE) [76]. MACE occurred in 0.8% with no plaque, 3.7% with nonobstructive disease, 27.6% with 1-vessel, 35.5% with 2-vessel and 57.7% with 3-vessel-disease.

No standardized score—such as the Agatston-Score for calcified plaques—exists for quantifying non-calcified plaques. Such a score which comprises the numbers of coronary segments with different morphology and amount of plaque is only used in studies [68,75]. In these, cardiac events are related to higher scores.

To conclude, rates of cardiovascular risk and MACE go hand in hand with the amount of plaque in the coronary arteries. Plaque morphology may play an important role, but it cannot be diagnosed on native calcium scans. A non-negligible number of individuals have non-calcified plaques in the absence of calcium, ranging from 4% to 25% according to selection of study population [68,78]. These patients might benefit most from CCTA for risk stratification over CAC although no data exist to prove that, and therefore CCTA is not recommended for that purpose.

## 11. Summary and Future Directions

CCTA is a very reliable diagnostic tool for proving and ruling out obstructive CAD. Still, and in spite of remarkable improvements in image quality due to progress in technology, there are factors such as severe obesity or calcifications that impede diagnostic accuracy. Studies have demonstrated that the type and amount of plaque are related to cardiac events independently of the remaining lumen diameter. Important prognostic implications have been proven especially for calcium scoring. Different types of plaque can be visualized by CCTA, and high-risk lesions being prone to acute coronary syndromes have been described. This ability of CCTA may be able to provide important prognostic information, particularly compared with ICA. However, it remains to be demonstrated whether specific treatment of morphological high-risk plaques on CCTA will translate into fewer future cardiac events and improvements in prognosis.
